# On the Front Line: Quantitative Virus Dynamics in Honeybee (*Apis mellifera* L.) Colonies along a New Expansion Front of the Parasite *Varroa destructor*

**DOI:** 10.1371/journal.ppat.1004323

**Published:** 2014-08-21

**Authors:** Fanny Mondet, Joachim R. de Miranda, Andre Kretzschmar, Yves Le Conte, Alison R. Mercer

**Affiliations:** 1 Department of Zoology, University of Otago, Dunedin, New Zealand; 2 INRA, UR 406 Abeilles et Environnement, Avignon, France; 3 AgroParisTech, Paris, France; 4 Department of Ecology, Swedish University of Agricultural Sciences, Uppsala, Sweden; 5 INRA, UR 546 Biostatistique et Processus Spatiaux, Avignon, France; Stanford University, United States of America

## Abstract

Over the past fifty years, annual honeybee (*Apis mellifera*) colony losses have been steadily increasing worldwide. These losses have occurred in parallel with the global spread of the honeybee parasite *Varroa destructor*. Indeed, *Varroa* mite infestations are considered to be a key explanatory factor for the widespread increase in annual honeybee colony mortality. The host-parasite relationship between honeybees and *Varroa* is complicated by the mite's close association with a range of honeybee viral pathogens. The 10-year history of the expanding front of *Varroa* infestation in New Zealand offered a rare opportunity to assess the dynamic quantitative and qualitative changes in honeybee viral landscapes in response to the arrival, spread and level of *Varroa* infestation. We studied the impact of *de novo* infestation of bee colonies by *Varroa* on the prevalence and titres of seven well-characterised honeybee viruses in both bees and mites, using a large-scale molecular ecology approach. We also examined the effect of the number of years since *Varroa* arrival on honeybee and mite viral titres. The dynamic shifts in the viral titres of black queen cell virus and Kashmir bee virus mirrored the patterns of change in *Varroa* infestation rates along the *Varroa* expansion front. The deformed wing virus (DWV) titres in bees continued to increase with *Varroa* infestation history, despite dropping infestation rates, which could be linked to increasing DWV titres in the mites. This suggests that the DWV titres in mites, perhaps boosted by virus replication, may be a major factor in maintaining the DWV epidemic after initial establishment. Both positive and negative associations were identified for several pairs of viruses, in response to the arrival of *Varroa*. These findings provide important new insights into the role of the parasitic mite *Varroa destructor* in influencing the viral landscape that affects honeybee colonies.

## Author Summary

Honeybees currently face a dramatic decline worldwide. The main honeybee parasite - *Varroa destructor* - plays a key role in these mortalities, since uncontrolled infestation inevitably results in the death of the colony. The pathological effects of *Varroa* infestations are partly attributed to the association of the mite with several honeybee viruses, primarily deformed wing virus (DWV). However the exact role that *Varroa* plays in the spread of honeybee viruses is still unknown. The recent arrival of *Varroa* in New Zealand provided a timely opportunity to gain insights into the complex relationship between bees, *Varroa* and viruses. Our data reveal that the different viruses have unique quantitative dynamics in relation to *Varroa* infestation, resulting in a shifting succession of virus infections that ultimately leaves DWV as the predominant infection. Assumption-free analysis shows consistent clustering of the data according to *Varroa*-infestation history, confirming a progressive change in the overall virus landscape co-incident with *Varroa* infestation. We also highlight possible interactions between several viruses. Our findings may have implications for the beekeeping industry, by highlighting the dynamic changes in the virus infections due to the arrival of *Varroa*, and how these are maintained.

## Introduction

The honeybee, *Apis mellifera*, plays an essential role in modern agriculture. In addition to honey production, honeybees provide critical ecosystem services, primarily pollination, for a large range of high-value agricultural crops [1]. However, during the last half-century honeybees have come under increasing stress [2] resulting in persistently increasing mortality rates of honeybee colonies worldwide [3]. The causes of this elevated mortality have yet to be fully unravelled. Changes in land use, crops and agricultural practices; new pesticides and more extensive pesticide applications [4]–[6]; increasingly intensive beekeeping; exotic parasites and the spread and increasing loads of honeybee pathogens [7], [8] have been proposed as major contributory factors to honeybee mortality.

Parasitism of bees by the mite *Varroa destructor* is currently considered to be one of the main causes of honeybee colony mortality worldwide [9], [10]. *Varroa* mites are obligatory ectoparasites that trigger both physical and physiological effects on individual honeybees, as well as impacts at the colony level. The mite's life cycle is closely tuned to, and highly dependent on the life cycle of the honeybee, as the mites reproduce in brood cells and feed on the haemolymph of their host [11], [12]. Mite infestation also has indirect pathological effects, including the spread and development of viral infections [13]–[16], which contribute significantly to the collapse of honeybee colonies [15], [17].

To date twenty-two viruses have been described in the honeybee [18]–[20], several of which have been linked to *Varroa* parasitism. These include many of the currently pre-dominant viruses; acute bee paralysis virus (ABPV), Kashmir bee virus (KBV), Israeli acute bee paralysis virus (IAPV), black queen cell virus (BQCV), chronic bee paralysis virus (CBPV), sacbrood virus (SBV), deformed wing virus (DWV) and *Varroa destructor* virus-1 (VDV-1) [19], [21], [22]. ABPV, KBV and IAPV on the one hand, and DWV and VDV-1 on the other hand belong to species complexes [23], [24] that include closely related virus species that share biological characteristics, such as transmission routes and pathology. Genetic variability that has an impact at the functional level, however still allows for distinct diagnoses of each species.

The degree to which viruses are linked to *Varroa* parasitism differs between viruses. The link is strongest for those viruses that are actively transmitted by *Varroa*, such as the DWV/VDV-1 and the ABPV/KBV/IAPV species complexes, but weaker for those viruses whose active transmission by *Varroa* is less certain, or absent, but that still benefit opportunistically from *Varroa*-weakened colonies. DWV is the virus most closely associated with *Varroa* infestation. In fact, the current prevalence, abundance and virulence of DWV appears to be almost entirely due to its transmission by *Varroa*: it was practically unknown prior to the arrival of *Varroa* in Europe, is even now undetectable in *Varroa*-free areas and gradually disappears from colonies with effective mite control. In areas where the mite is well-established DWV is usually the principal direct cause of *Varroa*-induced colony collapse [9], [10], [25]. However, in recently invaded areas, often the first viruses associated with *Varroa* are one of the ABPV-like viruses (ABPV, KBV, IAPV), which in *Varroa*-free areas are generally more prevalent than DWV, before these are gradually superseded by DWV [26]–[28]. This was the case in Europe during the 1980's [29], in the Americas during the 1990's [30], [31] and in New Zealand during the 2000's [32]. Both at intra- and inter-colony level, natural selection has apparently favoured transmission of an inherently low virulence virus (viruses of the DWV complex [24]) at the expense of an inherently high virulence virus (viruses of the ABPV complex [23]) [22], [33] through the primary requirement for any virus that the host (pupae, colonies) survives long enough to enable effective transmission (*Varroa* survival, dispersal). This also highlights the importance of mode of transmission for virus virulence [24]. Heavy *Varroa* infestations can lead to the development of clinical symptoms for a condition known as *parasitic mite syndrome*, the hallmark feature of which is an overt virus outbreak at the colony level [34].

The arrival of the mite in a new region coincides with overall increases in the prevalence and loads of most honeybee viruses [16], [19]. This has required a change in the conceptual framework of the relationship between *Varroa* and the honeybee, from a classical bilateral host-parasite relationship to a 3-way relationship that includes viruses. *Varroa*'s role in the spread of the different honeybee viruses depends on the nature of the relationship between the mite and the virus (active/passive vector; activator of infections; opportunistic secondary infections; augmentation of alternative transmission routes), which differs for each virus [23], [24].

Viral infections remain the least understood of honeybee pathologies due to the lack of mechanistic information about modes of virus spread and transmission [35]. This reflects in part, technical limitations such as difficulty in obtaining pure virus preparations and colonies that are free of *Varroa* and/or viruses.

**Figure 1:**
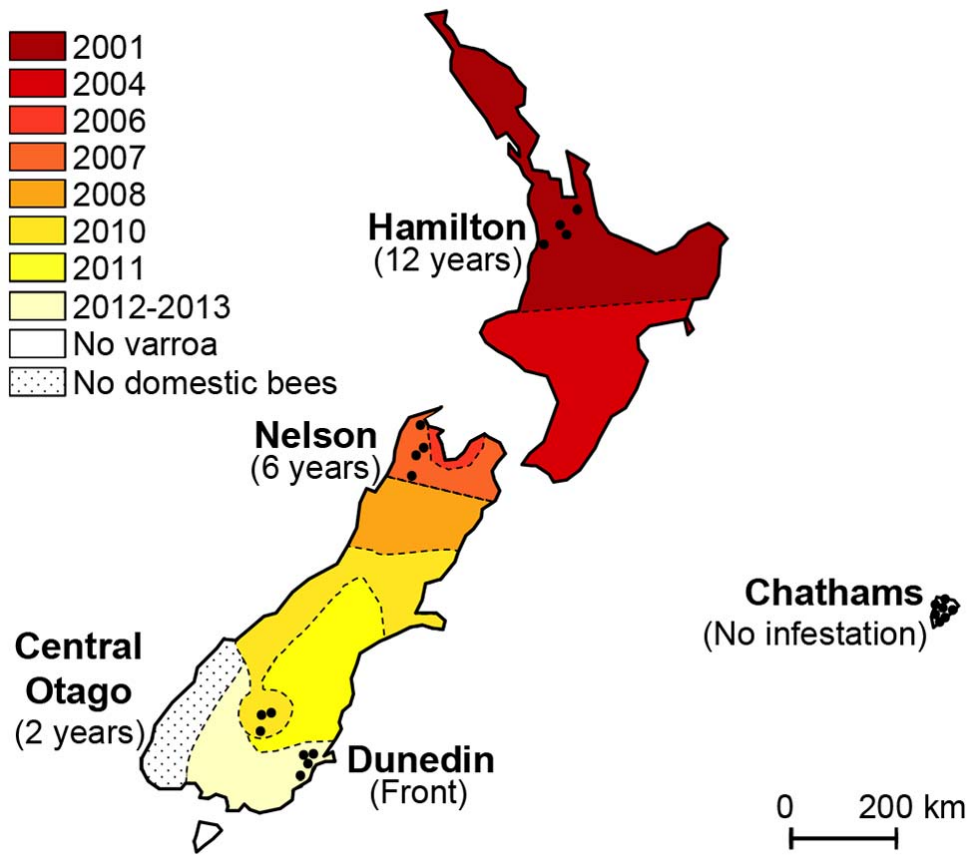
Map illustrating the spread of *Varroa* across New Zealand and the location of sampling sites. Colours indicate the date *Varroa* was first confirmed in each area. Shaded tones from dark red to light yellow show the progression of the front of *Varroa* infestation. Control regions where the mite had not yet been detected are presented in white. Black dots indicate the location of the apiaries sampled in each region. The sampling transect crosses the front of infestation.

In this study we took advantage of a naturally-occurring phenomenon that gave us access to a rare and potentially important set of samples. New Zealand has only recently been invaded by *Varroa* and still has an active infestation expansion front into currently *Varroa*-free areas. European honeybees were first introduced to New Zealand in 1839 [36]. Importations from many origins were subsequently recorded, until further importation was prohibited by the 1924 Apiary Act. Two sub-species of *Apis mellifera* are represented, mainly as hybrids, amongst New Zealand honeybees, *Apis mellifera ligustica* and *Apis mellifera mellifera* [37]. Until recently, New Zealand remained one of only a few *Varroa*-free countries in the world. However, in the year 2000, *Varroa destructor* was detected in managed colonies in the northern part of the North Island [38]. Reports suggest that the initial spread of *Varroa* lead to a 16% drop in colony numbers in the North Island [39]. Managed control programmes organised by the central Government helped slow the spread of the mite across the country but by 2006 *Varroa* was detected in the northern regions of the South Island, from where it continued its spread southwards. By the fall of 2013, the mite was considered to have infested most areas of mainland New Zealand, despite quarantine measures. When this study began in 2012, the mite-free areas included apiaries located South of Dunedin and on the Chatham Islands (Figure 1).

The aim of this study was to monitor the first stages of infestation by *Varroa* and its implications on the evolution of the complex interplay between bees, *Varroa* and viruses. Honeybee colonies were surveyed to assess the dynamic changes in the prevalence and titres of seven honeybee viruses since the arrival of *Varroa*. The expanding front of mite infestation was used as a proxy for years of *Varroa* infestation. The results reveal interesting and unexpected insights into the links between the prevalence, abundance and temporal changes in DWV, CBPV, BQCV, KBV and SBV in relation to the prevalence, abundance and length of *Varroa* infestation.

## Results

### *Varroa* prevalence and infestation rates

Two distinct *Varroa* parameters were recorded: *Varroa* prevalence, defined as the proportion of colonies in which mites could be detected, and *Varroa* infestation rate, defined as the number of phoretic mites per 100 bees for those colonies where mites could be detected. The sampling sites formed a transect that followed the historical front of expansion of the mite *Varroa destructor*. The number of years the parasite had been detected in a given area is indicated in Figure 1. A “region” was defined in this study as a geographical unit in which *Varroa* had either not been detected, or in which *Varroa* had been detected in commercially managed honeybee colonies at approximately the same time. In each apiary visited in this study, nine randomly-selected colonies were used to assess *Varroa* mite populations. The mite prevalence for each region was calculated as the proportion of colonies in which at least one mite could be recovered from three consecutive ‘sugar shakes’ performed on three samples of bees from each colony, with each sample containing approximately 300 adult bees, i.e. with an infestation rate of approximately 0.1% or greater.

**Figure 2:**
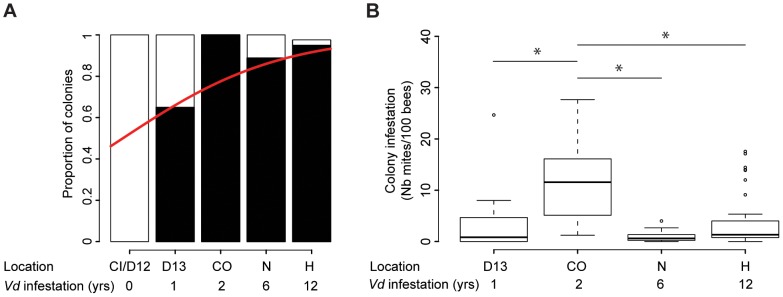
Quantitative analysis of the phoretic *Varroa* infestation. (**A**) Varroa prevalence. The proportion of colonies where mites could be retrieved (black) versus not retrieved (white) is presented in terms of the sampling site location and number of years *Varroa* had been detected in the area. A significant increase in *Varroa* prevalence along the sampling transect is symbolised by the red curve (GLMM, Z = 4.14, p<0.001, 27≤n≤39). (**B**) *Varroa* infestation levels according to the number of years of confirmed exposure to *Varroa*. Number of phoretic mites per 100 bees (27≤n≤39). Stars indicate significant differences between years of infestation (Pairwise post-hoc comparisons, p<0.01).

The prevalence of the parasite increased significantly along the transect, ranging from no infestation in the two regions where *Varroa* had not yet been reported, to between 85% and 100% prevalence in regions with 2 to 12 years of confirmed *Varroa* presence (Z = 4.146, CI_95%_ = (0.2533, 0.7077), p = 3.38.10^−5^ - Figure 2.A.). The prevalence obtained for apiaries with the longest infestation record are consistent with those found for colonies and apiaries in the Northern Hemisphere, sampled at a corresponding stage in the bee season (autumn) [12], [26], [40].

Differences in *Varroa* infestation rates across the five regions included in the sampling transect did not follow a linear trend (Z = −9.79, CI_95%_ = (−0.0678, −0.0454), p<2.10^−16^ – Figure 2.B). *Varroa* infestation rates were highest in the apiaries in their second year of confirmed infestation. Post-hoc comparisons using pairwise t-tests (adjusted for multiple comparisons using Bonferroni corrections) revealed significant differences between the colonies sampled after two years of infestation and all other confirmed periods of detection of *Varroa* (1–2 years: p = 4.10^−5^; 2–6 years: p = 1.2.10^−7^; 2–12 years: p = 3.6.10^−5^).

### Virus prevalence

**Figure 3:**
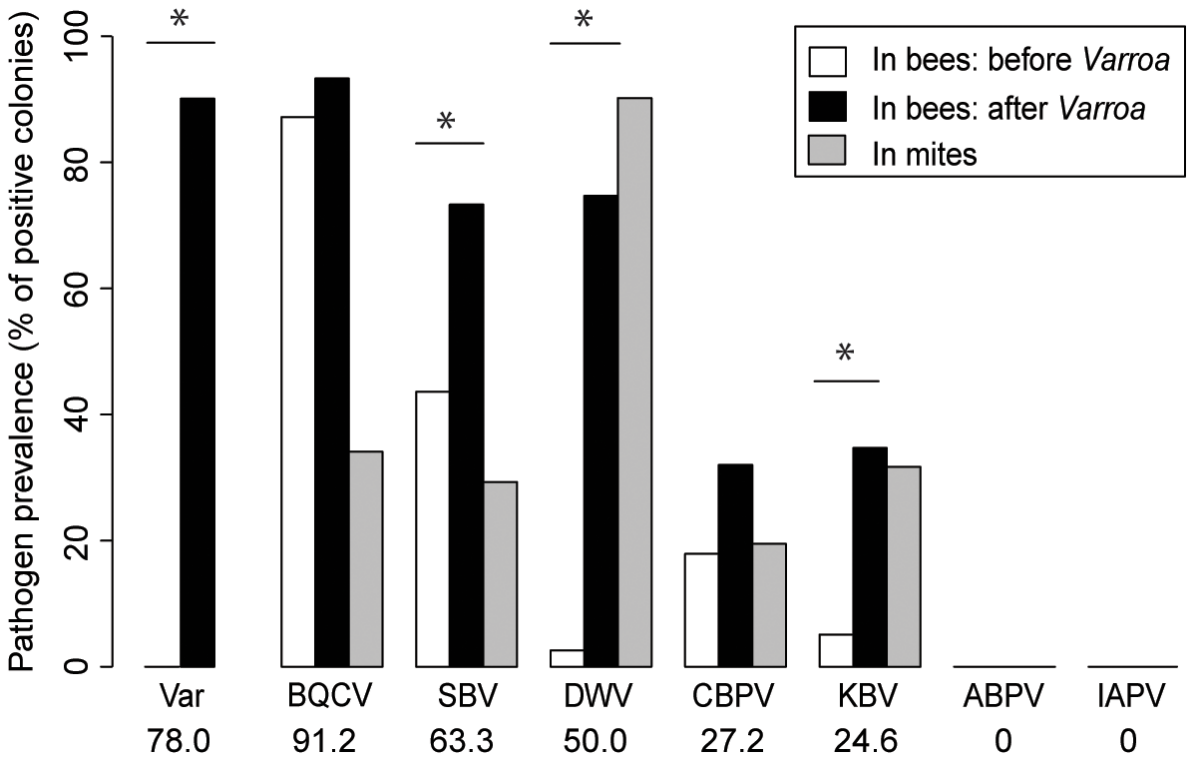
Honeybee virus prevalence across the *Varroa* front of infestation. Pathogen prevalence across the front of infestation, in bee samples and *Varroa* mite samples. The percentage of colonies assigned positive for each of the seven viruses monitored is compared between *Varroa*-free areas for bee samples (white bars, n = 39), *Varroa*-infested areas for bee samples (black bars, n = 75), and *Varroa* mite samples (grey bars, n = 34). Stars indicate significant differences between proportions (Chi-square, p<0.05). Viruses are presented in decreasing order of prevalence. The average pathogen prevalence in bee samples across all regions sampled is indicated on the x-axis below the pathogen acronym.

Of the seven honeybee viruses assessed in this study, five were detected repeatedly in honeybees from the colonies sampled (BQCV, SBV, DWV, CBPV and KBV). KBV was confirmed as the only representative of the ABPV/KBV/IAPV species complex [23], [32]; ABPV and IAPV were not detected. The average prevalence of the five viruses across the country, i.e. the proportion of colonies in which the virus was detected, ranged from 91.2% for the most prevalent virus (BQCV) to 24.6% for the least prevalent virus (KBV). DWV exhibited an average prevalence of 50% (x-axis percentages, Figure 3).

In bee samples, all 5 viruses had higher prevalence in the presence of *Varroa* than in the absence of *Varroa* (Figure 3). However the difference was significant only for DWV, SBV and KBV (BQCV: X^2^ = 0.57, p = 0.451; SBV: X^2^ = 8.52, p = 0.0035; DWV: X^2^ = 50.51, p = 1.2.10^−12^; CBPV: X^2^ = 1.89, p = 0.117; KBV: X^2^ = 10.54, p = 0.0016). DWV was the only virus that was not detected in areas where *Varroa* was not present (except for one colony in the Dunedin region in 2012), but it was detected frequently in infested areas. All five viruses detected in bee samples were also detected in mite samples (Figure 3 – grey bars). DWV displayed the highest prevalence for mite samples; 90% of the mite samples from *Varroa* infested colonies were positive for this virus. The colony-level prevalence of BQCV, SBV, CBPV and KBV as determined from mite samples, i.e. the proportion of colonies whose mite samples were positive for each virus, ranged from 17% to 36%. DWV was also the only virus that showed a higher prevalence in the mite samples than in the bee samples, although this difference was not significant (X^2^ = 3.13, p = 0.077). Contingency Chi-squared analyses were used to identify non-random associations, either positive or negative, between the prevalence of each virus in the two different sample types (mites and bees), as well as non-random co-infection of different combinations of viruses. For both DWV and KBV there was significant positive association between their detection in corresponding bee and mite samples (DWV: X^2^_1_ = 5.42, p = 0.02; KBV: X^2^_1_ = 13.82, p<0.001 - Table 1.), *i.e.* if the virus was detected in the bee sample then it was more likely to also be detected in the mite sample. For the other three viruses (BQCV, CBPV and SBV) there was no significant association between their detection in bee samples and corresponding mite samples (SBV: X^2^_1_ = 3.36, p = 0.067; BQCV: X^2^_1_ = 0.0065, p = 0.94; CBPV: X^2^_1_ = 0.093, p = 0.76).

**Table 1:** Contingency tables showing the contingency Chi-square values for non-random association of pairs of viruses in colonies in the *Varroa*-free areas (upper section, n = 39) and in the *Varroa*-infested areas (lower section, n = 75).

			*Varroa*-free				
	*X^2^_(1)_*		DWV	BQCV	CBPV	KBV	SBV
	DWV	Bee		1,27	0,71	1,76	0,017
		Mite					
	BQCV	Bee	0,33		0,25	0,0041	0,43
		Mite	3,42				
	CBPV	Bee	0,035	0,79		0,09	1,49
*Varroa*-infested		Mite	**5,22 ^(−)^**	**5,63 ^(+)^**			
	KBV	Bee	**4,80 ^(−)^**	2,44	2,42		0,067
		Mite	**4,38 ^(−)^**	3,69	3,69		
	SBV	Bee	**12,82 ^(−)^**	**11,84 ^(+)^**	1,40	0,0026	
		Mite	**6,37 ^(−)^**	**8,43 ^(+)^**	2,76	**4,05 ^(+)^**	

The contingency tables were derived through comparing the observed incidence of co-infection with the expected values derived from the individual prevalences of each virus. For significant non-random associations (bold; p<0.05) is also indicated whether the association is positive (+), i.e. a higher incidence of co-infection than expected, or negative (−), i.e. a lower incidence of co-infection than expected.

The increased individual prevalence of the viruses in *Varroa*-infested colonies naturally also increases the chance of detecting multiple virus infections in *Varroa*-infested colonies, compared to colonies from *Varroa*-free areas (t = 2.919, CI_95%_ = (0.5157, 2.6243), p = 0.0042). The number of virus species detected per colony (out of the seven honeybee viruses examined in this study) averaged 1.56+/−0.15 (n = 39) before the arrival of *Varroa*, but rose to 3.08+/−0.10 (n = 75) virus species per colony in apiaries in which *Varroa* had become established. This suggests that the presence of *Varroa* increases the number of viruses that can be detected in a colony.

The observed incidences of co-infection by two viruses were compared to predictions based on the individual prevalence of each virus, using contingency Chi-squares analyses, the results of which are shown in Table 2. These tests of non-random association between viruses were performed independently for the *Varroa*-free and *Varroa*-infested areas: in the latter case using the virus prevalence data from either the bee or the mite samples. For the *Varroa*-free areas there was no evidence of any positive or negative association between any of the viruses studied here. This pattern changes in the *Varroa*-infested areas, where there is evidence in the bee samples of negative (antagonistic) association between KBV & DWV and DWV & SBV, while in the mite samples there was also negative association between KBV & DWV, DWV & SBV, as well as between DWV & CBPV. In addition, in the bee samples there was positive association between SBV & BQCV, while in the mite samples there was also positive association between SBV & BQCV, as well as between SBV & KBV. There were not enough observations to assess higher order interactions, *i.e.* between three or four viruses.

**Table 2:** Contingency table analyses for virus co-prevalence in both bees and mite samples.

*X^2^_(1)_*	Bee-mite co-infection
DWV	**5,42 ^(+)^**
BQCV	0,0078
CBPV	0,09
KBV	**13,82 ^(+)^**
SBV	3,36

For the *Varroa*-infested region, separate comparisons were made for the virus prevalences and co-infection in bee samples and in mite samples (n = 41). The contingency tables were derived through comparing the observed incidence of co-infection with the expected values derived from the individual prevalences in bees and mites. For significant non-random associations (bold; p<0.05) is also indicated whether the association is positive (+), i.e. a higher incidence of co-infection than expected, or negative (−), i.e. a lower incidence of co-infection than expected.

### Virus titres in bee samples

In this section, we analysed the amounts (titres) of each virus species, with regards to the *Varroa* infestation rates. The relationship between the amounts (titres) of the five viruses, in bees and mites, and the *Varroa* infestation rates (mites per 100 bees) across the entire survey was initially analysed using principal component analysis (PCA). PCAs are multivariate analyses that identify the greatest sources of variation (principal components) in datasets combining multiple variables, without making any prior assumptions about the origins of the samples. The PCA approach allowed us to look neutrally for any trend in the complete quantitative data set, including both virus titres and mite infestation rates for each colony, independent from any assumption regarding the history of *Varroa* infestation of the colonies.

**Figure 4:**
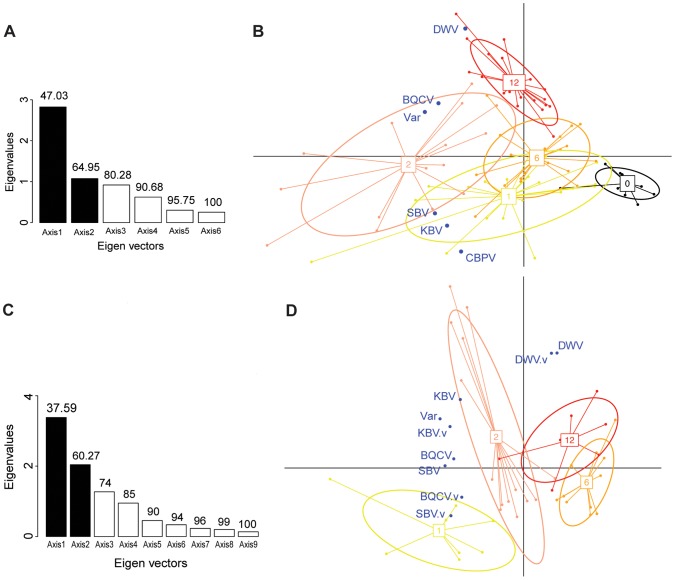
Principal component analyses of pathogen titres in honeybee and *Varroa* samples. (**A**) Barplot of the eigenvectors of the PCA performed on the variables measured in bees. Variables included in the Principal Component Analysis (PCA) are the titres of 5 viruses (DWV, BQCV, CBPV, KBV, SBV) and the *Varroa* infestation rate (Var). Numbers above each bar indicate the cumulative percentage of variability explained by the successive eigenvectors. The two principal eigenvectors, represented by black bars, correspond to the axes used to plot the colonies in Figure 4.B. (**B**) Scatterplot of colonies analysed by PCA for the titres of 5 viruses plus the *Varroa* infestation rates in bees (n = 191). The colony values for the two principal components are plotted, with each colony represented by a filled circle. The colonies are clustered by colour and bound by an ellipse according to the number of years since the first detection of *Varroa*, indicated by the number located at the centre of gravity of each ellipse. The ellipse covers 67% of the samples belonging to the cluster. The colour code is the same as for Figure 1. (**C**) Barplot of the eigenvectors of the PCA performed on variables measured in bees and in *Varroa*. Variables included in the PCA are the titres of 4 virus species in bees (DWV, BQCV, KBV, SBV), titres of 4 virus species in *Varroa* (DWV.V, BQCV.V, KBV.V, SBV.V) and the *Varroa* infestation rates (Var). The numbers above each bar indicate the cumulative percentage of variability explained by the successive eigenvectors. The two principal eigenvectors, represented by the black bars, correspond to the axes used to plot the colonies in Figure 4.D. (**D**) Scatterplot of colonies analysed by PCA for virus titres in bees and mites plus the *Varroa* infestation rates (n = 83). The colony values for the two principal components are plotted, with each colony represented by a filled circle. The colonies are clustered by colour and bound by an ellipse, according to the number of years since the first detection of *Varroa*. Each ellipse covers 67% of the samples belonging to the cluster.

**Figure 5:**
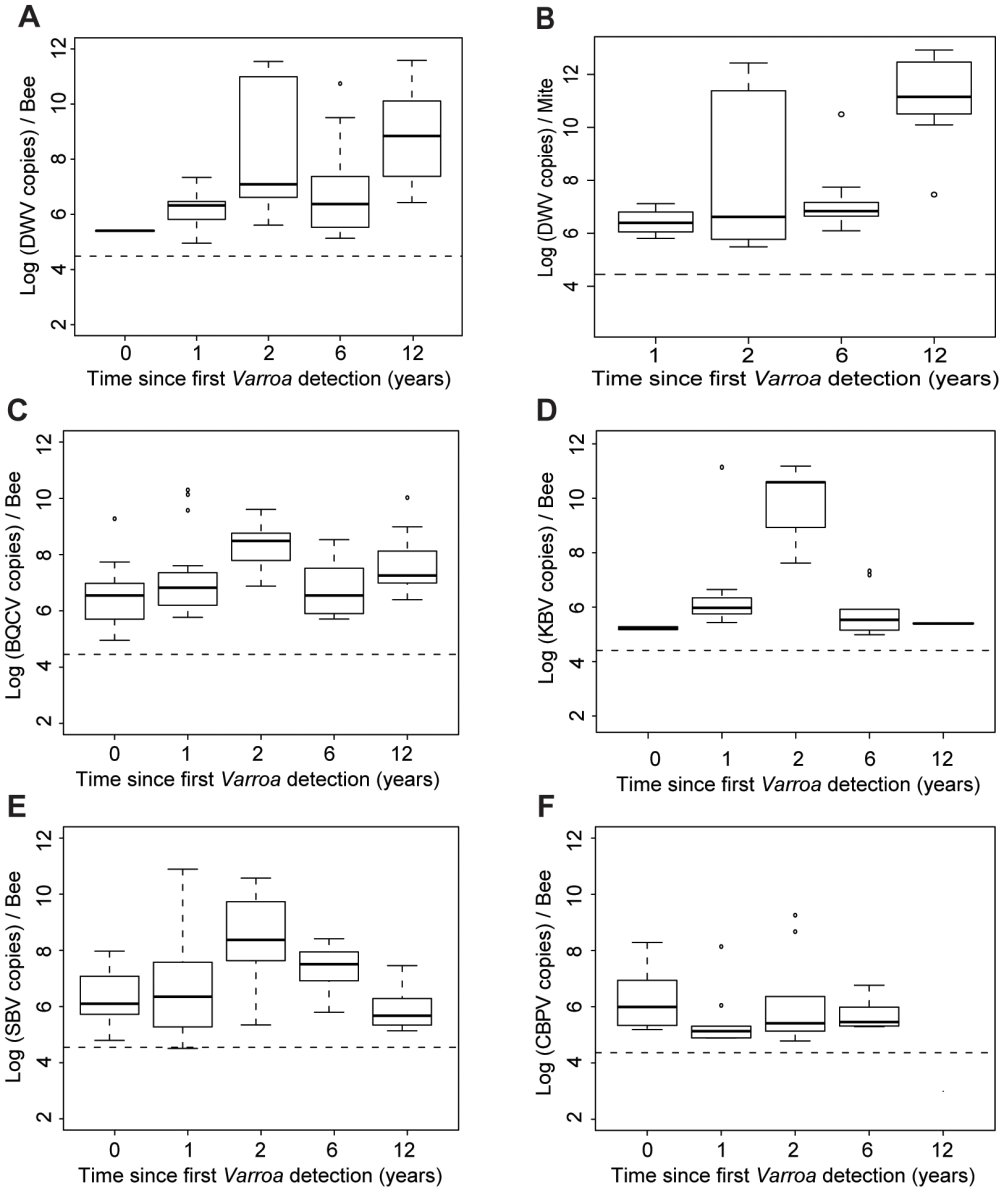
Virus titres in honeybees and *Varroa* mites along the *Varroa* front of expansion. (**A**) DWV titres in bees (Log_10_ DWV copies/bee) according to the number of years of exposure to *Varroa*. A significant increase in the level of viral infestation was detected along the sampling transect (GLMM, t = 3.78, p<0.001, 30≤n≤41). (**B**) DWV titres in *Varroa* (Log_10_ DWV copies/mite) according to the number of years of confirmed exposure to *Varroa*. A significant increase in the level of viral infestation was detected along the sampling transect (GLMM, t = 4.55, p<10^−5^). (**C**) BQCV titres in bees (Log_10_ BQCV copies/bee) according to the number of years of exposure to *Varroa*. A significant increase in the level of viral infestation was detected along the sampling transect (GLMM, t = 3.35, p<0.001, 30≤n≤41). (**D**) KBV titres in bees (Log_10_ KBV copies/bee) according to the number of years of exposure to *Varroa*. (**E**) SBV titres in bees (Log_10_ SBV copies/bee) according to the number of years of exposure to *Varroa*. (**F**) CBPV titres in bees (Log_10_ CBPV copies/bee) according to the number of years of exposure to *Varroa*.

The first PCA analysis included 6 variables: the titres of each of the 5 viruses in the bee samples plus the *Varroa* infestation rate. The barplot of the eigenvalues (Figure 4.A.) suggests that the two principal components explain about 65% of the overall variability of the data. The scatterplot of the colonies analysed for pathogen titres in bees, when projected on the plan formed by the two first eigenvectors, showed a clear clustering of the colonies according to the number of years of confirmed exposure to *Varroa* (Figure 4.B.). Interestingly, along the DWV vector direction, the colony clusters segregated according to the increasing number of years of *Varroa* detection (Figure 4.B.), which mirrors the trend of DWV titre increase with years of *Varroa* infestation (Figure 5.A.). This was not true for the *Varroa* infestation rate vector direction, which is consistent with the observation that the *Varroa* infestation rates did not exhibit a linear trend along the entire sampling transect, but rather a peak at the two-year mark, followed by a decrease (Figure 2.B.). The clusters, delineated by ellipses in Figure 4.B., move in the direction of the *Varroa* infestation rate vector for years 0-1-2 before reversing direction in year 6 and moving in an orthogonal direction year 12.

To investigate further the influence of *Varroa* during its initial phase of establishment on the titres of the five viruses analysed in this study we postulated that the sampling transect could be considered a proxy of the number of years of *Varroa* detection for each region.

The titres of the different viruses in relation to the time since *Varroa* mites were first detected are shown in Figure 5. For most viruses there is a gradual increase towards peak after 2 years of *Varroa* detection, following the peak in *Varroa* infestation rate (Figure 2.B.). After this, both KBV and SBV decline, again following the *Varroa* infestation pattern, while DWV continues to increase. The pattern for BQCV is somewhere in between that of DWV on the one hand, and SBV & KBV on the other hand. The titres of CBPV do not present noticeable variations along the sampling transect. DWV titres in adult bees increased significantly with the duration of *Varroa* infestation (t = 3.78, p<0.001 – Figure 5.A.). A similar progressive increase, though less steep, was observed for BQCV (t = 3.35, p<0.001 – Figure 5.C.). KBV and CBPV almost disappeared after 12 years of *Varroa* infestation, while SBV returned to similar levels as observed pre-*Varroa* infestation (Figure 5.D–F).

The dynamics of changing pathogen loads along the front of mite infestation were revealed effectively also by comparing the temporal patterns of the *Varroa* infestation rates and virus titres (Figure S1.A.). For each pathogen (*Varroa* and viruses), colonies were grouped according to the number of years of *Varroa* exposure and average titres or infestation rates were calculated for each of the five resulting groups (0, 1, 2, 6 and 12 years of *Varroa* exposure). The vectors containing these five average values for each virus or *Varroa*, which represent the temporal pattern of each pathogen, were then compared using correlation analyses. This allowed us to assess to what degree the dynamic changes in the titres of each virus along the *Varroa*-front of infestation mirrored the change in the *Varroa* infestation rates. Interestingly, the patterns for BQCV and KBV titres were positively correlated to the pattern of the levels of *Varroa* (BQCV: S = 2, p = 0,042, rho = 0.9; KBV: S = 4, p = 0.042, rho = 0.9). By contrast, the CBPV, SBV and, most strikingly, the DWV titre patterns did not show any direct correlation with the pattern of *Varroa* infestation rates (SBV: S = 10, p = 0.23, rho = 0.5; CBPV: S = 26, p = 0.74, rho = −0.3; DWV: S = 10, p = 0.23, rho = 0.5).

Possible relationships between *Varroa* infestation rates and virus titres were investigated also by regression analysis, using the entire *Varroa*-infestation data set, without taking into account the number of years of exposure to the mite (Table 3). Significant linear relationships were detected between *Varroa* infestation and the BQCV and KBV titres, and to a lesser extent the SBV and DWV titres (Figure S1.B.). No relationship emerged between mite infestation and CBPV titres.

**Table 3:** Results of the regression analyses.

Virus titres in bees	*Varroa* infestation rates				Virus titres in mites			
	*F*	*df*	*p*	*r^2^*	*F*	*df*	*p*	*r^2^*
DWV	17.63	1,54	<0.001	**0.25**	27.81	1,28	<0.001	**0.5**
KBV	23.16	1,24	<0.001	**0.49**	4.03	1,10	0.072	0.29
BQCV	38.93	1,68	<0.001	**0.36**	2.58	1,12	0.13	0.17
SBV	16.98	1,53	<0.001	**0.24**	0.031	1,10	0.86	0.003
CBPV	0.070	1,23	0.79	0.003	-	-	-	-

Regression analyses were performed to compare the overall titres of each of the five viruses measured on bee samples (Virus titres in bees) with either the overall level of infestation by *Varroa* (*Varroa* infestation levels) or with the overall titres of each of the five viruses measured on *Varroa* samples (Virus titres in mites). Regressions were run on Log_10_ transformed data. R-squared with associated p-values<0.05 are indicated in bold. All significant regressions presented positive correlations (r>0).

The PCA indicates that *Varroa* infestation rates and DWV titres are not likely to be highly correlated. Consistent with this conclusion, the regression analysis, which was conducted on the entire dataset also, but which did not isolate the number of years of exposure to *Varroa*, shows a relatively weak correlation between these two variables (r^2^ = 0.25). Correlation analysis, in which number of years of exposure to *Varroa* is accounted for, shows that DWV patterns (changes in virus titres along the front of infestation) do not show any correlation with the pattern of *Varroa* infestation rates. These slightly differing conclusions are likely to be explained by the dynamic processes occurring along the front of infestation. In particular, the DWV titres and mite infestation rates are only closely related during the first few years of mite establishment with both exhibiting significant increases, after which the DWV epidemic progresses in part independently of the *Varroa* infestation rates, thus uncoupling any relationship in later years of infestation.

### Virus abundances in bee and *Varroa* samples

From the previous section, we concluded that the *Varroa* infestation rate and/or *Varroa* infestation history seems to influence the titres of some of the viruses found in adult bees. To investigate these relationships further, we analysed titres of viruses detected in bees alongside a new set of variables related to *Varroa* – virus titres measured in mites. Our aim was to determine if introducing these additional variables would alter the relationships established between the *Varroa* infestation rates and history, and the virus titres measured in bees.

To this end, a second PCA analysis was performed using colonies from which both honeybee and *Varroa* mite samples were available. In this case, 9 variables were used to run the PCA: the *Varroa* infestation rates and the BQCV, DWV, SBV and KBV titres from bee samples, plus the BQCV, DWV, SBV and KBV titres from mite samples. The CBPV titres were not included as a separate variable because of the low number of mite samples that detected positive for this virus.

The barplot of the eigenvalues suggests that the two principal components explain about 60% of the overall variability in the data (Figure 4.C.). The scatterplot of the colonies analysed for pathogen titres both in bees and in mites (Figure 4.D.) shows a similar clustering of the data points to the scatterplot of colonies analysed for bee samples only (Figure 4.B.). Interestingly, the DWV titre variable in bees co-localised perfectly with the DWV titre variable in mites (Figure 4.D.), suggesting the DWV titres in bees and mites are linked, or subject to similar regulating processes. As with the first PCA, a clear region-related gradient based on DWV titres in mites could be seen (Figure 4.D.). This tendency was confirmed by the pattern of DWV titres in mites along the sampling transect (Figure 5.B.).

The DWV titre development over the years in mites is similar to the DWV titre development in the bee samples, but more pronounced, especially towards the later years. A significant increase in the number of DWV genomic copies in *Varroa* could be identified as the number years of *Varroa* exposure increased (t = 4.55, p<10^−5^ – Figure 5.B.). This pattern was particularly interesting with regards to the trend highlighted for DWV titres in bee samples (Figure 5.A.). In addition, a regression analysis revealed a highly significant linear relationship between DWV titres in bees and DWV titres in mites (F_1,28_ = 27.81, p = 1.313.10^−5^ – Figure S1.C.). Most importantly, the degree of correlation between both variables was quite high (r^2^ = 0.498, Table 2), suggesting a possible functional link between DWV titres in bees and in *Varroa*. No correlation was identified between titres of BQCV, KBV or SBV in bees and in mites (Table 3).

## Discussion

### New Zealand as a model to study the impact of *Varroa* spread in honeybee colonies

This study investigated the influence of *Varroa* on the spread of seven honeybee viruses during the initial and medium-term phases of establishment of the parasitic mite in New Zealand. The relatively recent arrival of the mite in this country and its rapid spread across the two main islands provided a unique opportunity to gain insights into the interactions between honeybees, *Varroa* and different honeybee viruses. Our study suggests that within honeybee colonies in New Zealand, the viral landscape has changed dramatically since the arrival of the mite, around 10–15 years ago. Significant colony losses due to *Varroa* infestation have been reported during this period [39].

As revealed in a similar survey performed in the Hawaiian archipelago [27], changes to the viral landscape in response to the spread of *Varroa* are virus specific, *i.e.* each virus responded in a unique way to the arrival, establishment and persistence of the mite. Multivariate analysis (PCA) revealed that viral titre data obtained in our study fall into five distinct clusters, with each cluster containing colonies with the same history of *Varroa* infestation. We cannot rule out the possibility, however, that the observed clustering of data could be due to factors other than the number of years of *Varroa* exposure. Geographical parameters, such as latitude and altitude, and differences in climate, or in beekeeping practises between regions could also contribute to regional differences that might affect viral titres [2]. Although the study was designed to reduce, as far as possible, any bias introduced by such factors, through variations in the climatic conditions, altitude and management practises between different apiaries within each region, the ultimate proof of whether the *Varroa* expansion front can be reliably used as a proxy for time since infestation will be when these data are compared directly with multi-year analyses of the same apiaries. In the present study, apiaries within one region (Dunedin) were sampled twice, once in 2012 and once in 2013, after *Varroa* was detected in this region. Results show an increase in DWV titres in bees in 2013, similar to the increase in DWV seen with increasing years of mite infestation. This supports the hypothesis that the differences in virus titres in the different regions are most likely a consequence of their *Varroa* infestation history, and not a consequence of regional environmental differences.

### The arrival of the *Varroa* mite increases cases of multiple viral infections

Of the viruses examined in this study BQCV was the most prevalent virus, and its prevalence in New Zealand is very similar to its reported occurrence in other regions of the world [26]. SBV was also found to be very widely distributed across New Zealand, following trends reported worldwide [18], [41]. Interestingly, DWV was found in only 50% of the colonies examined in this study. This might reflect the recent arrival of the mite in New Zealand because in the areas where *Varroa* had not yet been detected, only one single DWV-positive colony was recorded. In the regions that had been infested with *Varroa* the longest, DWV prevalence reached 85% to 100%, which is in accordance with data collected in the US, Austria and France [19], [26], [42]. This dichotomy between near-absence of DWV in Varroa-free areas and near-ubiquity of DWV in Varroa-infested areas is in complete agreement with the history of this virus, and its close relationship with ectoparasitic mites [24].

All five of the virus species detected in bee samples could also be detected in mites, confirming previous findings for DWV, KBV, CBPV, BQCV and SBV [14], [43]–[51].

Our findings confirm also that honeybee and *Varroa* populations are frequently co-infected with multiple virus species [26], [44], [48], [52]–[54]. Such multiple infections create opportunities for interactions between viruses and other honeybee pathogens, which are likely to have cumulative (additive) and possibly also synergistic (interactive) effects on honeybee health at both individual and colony level [55]. Such synergistic interactions have been reported between the fungal pathogen *Nosema* and CBPV with respect to virus replication [56] and between *Nosema* and BQCV with respect to virus infectivity [57] although more recent research suggests that the effect of *Nosema* and BQCV on longevity are additive rather than synergistic, with *Nosema* by far the more damaging partner [58], and with drones much more susceptible to these pathogens than worker bees. Interactions of this kind are known to create unpredictable epidemiological effects in plants and other animal models [59], [60].

### *Varroa*-virus association in honeybees: a trade-off between virulence and transmission?

Virus-virus interactions have been studied both theoretically and experimentally in honeybees with respect to the displacement of ABPV (or its relatives KBV and IAPV) by DWV as the primary *Varroa*-associated virus during the early stages of *Varroa* establishment in new colonies or regions. This has been the pattern during the first invasion of *Varroa* into Europe [13] the Americas [30], [34], [61] and also New Zealand [32]. Both these complexes of viruses are efficiently transmitted by *Varroa*, and even replicate in the mite [23], [24], [62]. However, the ABPV-complex viruses are excessively virulent when transmitted by mite to developing pupae, causing them to die prior to emergence, thereby entombing the mite and its progeny. By contrast, DWV, a much less virulent virus, allows the mite to complete reproduction on the pupae. Often the resulting adults are still largely functional despite infection, at least during the early stages of the DWV epidemic. These factors help the honeybee colony survive, which in turn benefits the mite's chances of dispersal. This difference between ABPV and DWV is a strong selective force in favour of DWV, both at individual bee (pupae) and colony levels (winter survival), resulting in a rapid displacement of the ABPV-complex viruses with DWV during the first 2–3 years of infestation, as part of a natural succession driven by natural selection through virus transmission [15], [63]. A similar pattern can be observed in the data presented in this study, with titres of KBV (the only representative of the ABPV-complex species found in New Zealand [32]) peaking two years after initial infestation, before disappearing from the colonies entirely, and DWV gradually taking the place of KBV as the primary *Varroa*-transmitted virus. When *Varroa* first arrived in New Zealand, near Auckland, no DWV could be detected in heavily infested colonies while KBV was highly abundant [32]. Currently KBV is practically absent from this area, while DWV is ubiquitous and abundant.

For pathogens such as KBV, with a high virulence that is directly coupled to *Varroa*-mediated transmission, there may be a more direct relationship between virus titres and *Varroa* infestation dynamics than DWV, whose effects and quantitative dynamics develop more indirectly in relation to *Varroa* infestation, through the progressive development of an epidemic [28]. Because high virulence viruses like ABPV and KBV are likely to kill the pupa or adult bee on which they reproduce or live respectively, they may also exert a regulatory role on the *Varroa* life cycle. Interestingly, a strong negative association was found between KBV and DWV prevalence in this study, subsequent to the arrival of *Varroa*, supporting the displacement hypothesis outlined above. Similar processes may affect the relative prevalence and abundance of other viruses.

SBV was the virus whose prevalence was most frequently affected, either positively or negatively, by other viruses, especially in the presence of *Varroa* (Table 1). Previous studies also showed that in the presence of *Varroa*, SBV titres were often correlated to those of other viruses, even though no direct relationship between SBV and *Varroa* could be found [64]. It may be that this virus is particularly sensitive to the pathological changes caused by *Varroa*, even though it is not directly affected by the mite. This could explain the conflicting evidence for its association with *Varroa* infestation. SBV causes a disease of open brood, whose removal is affected by colony strength. SBV also affects adult bees, causing a marked aversion to pollen (consumption and foraging), which in turns leads to reduced brood care, earlier (nectar) foraging and reduced adult lifespan [52], [65], [66]. These are all important factors in general colony health, which are also affected by *Varroa* and the virus epidemics it transmits.

CBPV is transmitted by contact and therefore a disease associated with aggression (robbing) and overcrowding [67]. *Varroa* and its virus epidemics tend to reduce colony size and crowding but this also increases the chances of robbing attacks later in the season. In the absence of clear evidence for direct CBPV transmission by *Varroa*, this could lead to conflicting evidence of indirect association of CBPV with *Varroa*, depending on which dynamic predominates at any one time. In these studies, there was no evidence of any association of CBPV with *Varroa* infestation.

### *Varroa* is closely associated to several honeybee viruses in the early stages of infestation

The titre variations of two viruses in this study - KBV and BQCV – are strongly related to the variations in *Varroa* infestation rates. Across the entire survey, the titres of four viruses - KBV, BQCV, DWV and SBV – were positively correlated to the *Varroa* infestation rates. In addition, a striking increase in the prevalence of DWV, SBV and KBV was associated with the arrival of *Varroa* in New Zealand apiaries. No significant increase in prevalence was observed for BQCV, although this could be because the pre-*Varroa* prevalence of this virus had already reached almost 90%.

Despite the near ubiquitous presence of BQCV and SBV in honeybee colonies, there is as yet no conclusive proof that the mite acts as an active vector of either virus. The prevalence and titres of both these viruses in bees seem to be largely independent of *Varroa* infestation [16], [64]. Although there is plenty of evidence of an active transmission of ABPV and related viruses (KBV, IAPV) by *Varroa* [23], [68], [69], the study most similar to the present one, involving recent *de novo Varroa* infestation of the Hawaiian islands, found no association between *Varroa* infestation history and the prevalences or titres of these viruses [27]. However, earlier work suggested that the feeding activity of the mite could induce or activate the replication of pathological infections in bees [13], [14], [52], and that transmission of KBV by the vector *Varroa* could rely on immunosuppression mechanisms [48]. Because SBV, KBV and BQCV could already be detected in colonies across New Zealand before the arrival of *Varroa*, it is possible that *Varroa* activated, directly or through immunosuppression, some of these pre-existing covert viral infections, leading to an increased prevalence of these viruses even without active transmission by *Varroa*.

Several studies provide indirect evidence for the ability of *Varroa* to limit the immune response of bees, through alterations of immunity related pathways [40], [70], [71], such as the NF-κB signalling pathway, with as direct consequence an increased proliferation of viruses [40]. Thus it is possible that the effect of *Varroa* infestation on general honeybee colony health and strength could lead to the proliferation of some virus species as secondary infections.

### Mite infestation levels of a colony may not reflect DWV titres in honeybees

Many studies have shown that *Varroa* is an effective vector for DWV by positively affecting the number of DWV viral copies found in bees or honeybee colonies [14], [48], [72]–[74]. This is especially evident for studies with individual bees or pupae infested by mites [14], [48], [72], [75]. However, at colony level the link between *Varroa* infestation rates and DWV titres is affected by the dynamics of the DWV epidemics, both in infested bees and non-infested bees, which lags behind the population dynamics of the mite [74]. These vector-virus epidemic dynamics are unique to individual colonies, or even to different times during the season, causing a disruption of the correlation between colony mite infestation rates and DWV titres, when assessed over many colonies in different stages of the epidemic [72]. Locke et al [64] and Francis et al [73] found strong correlations between *Varroa* infestation rates and DWV titres in colonies undergoing various treatments for mite control (0.67≤r≤0.87). Using pupae naturally and artificially infested with *Varroa destructor*, Shen et al [48] showed a level of correlation between the number of mites parasitising a pupa and pupal DWV levels of less than 50% (r = 0.42). Di Prisco et al [72] looked at the effect of rearing newly emerged bees with different levels of *Varroa* for 7 days. They identified a strong correlation between high *Varroa* levels and DWV titres, but only for weak colonies. The correlation was not observed in strong colonies, suggesting that other factors in addition to mite infestation levels contribute to regulating DWV titres in honeybee colonies. Interestingly, Hedtke et al [74], who monitored colonies over a 6 year period, found that DWV infection in autumn correlated to *Varroa* levels observed in summer only, suggesting that there is a lag between dynamic shifts in the levels of the two pathogens.

Naturally, active *Varroa* control by beekeepers, over multiple seasons, would also disrupt any correlation between *Varroa* infestation rates and the virus epidemics it has helped initiate and perpetuate. In the present study this influence was minimized by strict adherence by the cooperating beekeepers to a national *Varroa* management strategy that included both spring and autumn treatments in all the *Varroa*-infested regions covered in this study. Despite such active *Varroa* management, the virus epidemics progressed rapidly, testifying to the influence of the alternative transmission routes to sustain the inertia of the epidemics through periods with low *Varroa* infestations. Different viruses may also have different capacities in this respect, leading to the different patterns of virus succession and epidemics mentioned above. The correlation between *Varroa* infestation rates and DWV titres in honeybee colonies explained about one quarter of the overall variability within the data (r^2^ = 0.24). This is consistent with the argument above that this global correlation is affected by other factors, such as the stage of the epidemics in different colonies, overall colony strength and the presence, transmission and damage caused by other viruses [63], [76]. Depending on these colony-specific epidemic factors, colonies can exhibit low levels of DWV even with significant *Varroa* infestation rates, and *vice versa*.

### Is *Varroa's* ability to actively host DWV a key factor affecting DWV dynamics in honeybees?

PCA analyses performed in this study on bee samples alone, and on bee and mite samples in combination generated a similar outcome; there was a clear clustering of data according to the number of years of confirmed exposure to *Varroa*. In addition, a strong correlation was identified between the DWV titres in mites and bees, which has also been observed previously, at both colony and individual bee level [64]. Results from the PCA and regression analyses suggest that mites play an important role in the transmission and development of DWV titres in bees [47]. This conclusion is strongly supported by the ability of DWV to replicate also in mites [14], [24], [62], [77], which allows the virus epidemic to build through the mite population as well as the bees. However, this replication competence only applies to a fraction of the mite population, since within any one colony the majority of mites seem to acquire and transmit the virus mechanically [47], [49], [62]. This incomplete and variable DWV replication competence of *Varroa*, as well as the link between DWV symptom development in bees and replication competence in mites [62], may be another factor disrupting the correlation between *Varroa* infestation rates and DWV titres in colonies. DWV replication competence in mites naturally leads to higher DWV titres in mites, with consequently larger inoculation titres to pupae, leading to a greater chance of overt symptoms in the emerging adult bee stage [78]. Another logical consequence is that evidence of DWV replication is particularly strong in those mites with very high DWV titres (10^10^–10^12^ genome equivalents per mite) [14], [62], [77], although such high titres could also be acquired passively, directly from highly infected pupa. Therefore also the relationship between DWV replication in mites, virus titres, and symptoms is a dynamic one, changing as the epidemics develops. Such high DWV titres were detected frequently in mite samples from the New Zealand colonies, especially in the region that had been infested with *Varroa* for more than 10 years, where the epidemics had been established the longest.

Reports of colony losses attributed to *Varroa*, together with signs of deformed-winged bees, have been most frequent amongst the professional beekeeping community in the region of New Zealand with the longest history of *Varroa* infestation, and with the highest DWV titres in the mite population. These pathological manifestations at colony and individual level could be at least partly due to an active replication of DWV in the mites, which would maintain sufficiently high DWV titres in emerging bees for wing deformities to present themselves regularly enough to be observed by beekeepers. Such observations along the *Varroa* front of expansion support the view that the ability of the mite to replicate DWV is the key factor driving DWV dynamics and its interactions with both bees and *Varroa* mites.

Results of this study strengthen the idea that the multiple virus infections in honeybees interact to create a dynamic and turbulent pathological landscape that peaks 2–3 years after *Varroa* infestation, after which it settles into a more stable and predictable pattern [27] and that these viruses individually and in concert play an important part in the survival or mortality of honeybee colonies infested by *Varroa* [14], [46], [75]. The ability of mites to persistently transmit viruses such as DWV appears as a crucial prerequisite to *Varroa* pathogenicity, and this may be due to the existence of virus strains with differing virulences for different infection scenarios [27]. Future research will focus on unravelling the mechanisms that are at the evolutionary basis of the bee-*Varroa*-virus complex [79]. Such knowledge is essential to understand the link between virus dynamics and the development of pathological signs that can ultimately lead to honeybee colony collapse.

## Materials and Methods

### Experimental design

*Varroa destructor* was first detected in New Zealand in 2001 near Auckland on the North Island and has since gradually extended its range southwards, crossing Cook Straight to the South Island in 2006 (Figure 1). The expansion front is currently located near Dunedin on the South Island. The movement of the *Varroa* expansion front has been carefully monitored during the last decade, such that the history of the expansion front is an accurate measure of the number of years of *Varroa* infestation for colonies in the areas behind the expansion front. The experimental design of the study was to sample honeybee colonies throughout the North and South Islands and monitor quantitative changes in the relationship between bees and their viruses since the arrival of *Varroa*, using the mite expansion front as a proxy for years of *Varroa* infestation. The Dunedin region, where the expansion front was located prior to the study, was sampled twice; first when it was ahead of the expansion front and mite-free and again after *Varroa* had invaded the area. Five regions were included in the study, contributing six *Varroa*-infestation scenarios at the time of sampling (2012): 11 years (Hamilton), 5 years (Nelson), 2 years (Central Otago) after *Varroa* invasion; the expansion front (Dunedin) before (2012) and after (2013) *Varroa* invasion and a *Varroa*-free region (Chatham islands). Colonies from the Chatham islands as well as from the first sampling year in the Dunedin region were included as a control to examine viral levels in the absence of mite infestation.

### Sampling and *Varroa* management

Four professional beekeepers in each of the five regions examined contributed each at least one permanent apiary site to the study, resulting in a total of 22 apiaries from across New Zealand. To avoid any possible effect due to the latitudinal organisation of the sampling transect, apiaries within one region were located in very different types of landscapes (forest, plains, plateau), altitudes (lowland versus high-country), rainfall and humidity exposures (inland versus coastal), and potentially beekeeping practises (each apiary belonged to a different beekeeper). The apiaries were also representative of all apiaries managed by the beekeeper, both in terms of apiary size and *Varroa* infestation, and had been in use for more than two years. The mainland apiaries had an average of 18±4.4 hives while the Chatham Island apiaries had an average of 4.6±0.8 hives per apiary.

The *Varroa* management strategies of New Zealand apiaries consist of two treatments each year, once in the spring and once in autumn. Beekeepers participating in this study used a variety of commercially available products, primarily Apivar, but also Bayvarol, Apistan, Apiguard, oxalic acid and Thymovar, all of which provide similarly adequate *Varroa* control [12].

Despite their relatively recent history of *Varroa* infestation, the colonies in the Dunedin and Central Otago regions had also been treated according the standard New Zealand strategy since *Varroa* was declared present in these regions, i.e. for one and two years respectively. In order to standardize the sampling protocol for all regions included in this study, sampling took place just before beekeepers applied the autumn *Varroa* treatments to their colonies [64]. This timing was chosen to ensure that any relationship between virus titres and mite infestation rates could be validly established, and not be influenced by the removal of mites through the autumn miticide treatment, which is the more important of the two treatments each season. Furthermore, autumn is also the season when both the mite infestation rates [12] and the titres of most honeybee viruses are at their highest [32], [44], [52], [80], allowing for the best possible resolution of their mutual relationship.

Nine colonies were selected randomly from each apiary site for *Varroa* infestation rate analysis, with hives located at the ends of rows excluded from inclusion to avoid potential margin effects. The phoretic *Varroa* mite infestation rate was determined on site using the ‘sugar shake’ method [81] on a sample of approximately 300 bees, collected from a frame containing uncapped brood and honey storage. The infestation rates were used to select five colonies for assessment of virus levels, excluding both the two colonies with the highest and the two lowest infestation rates from further participation in the experiment. This procedure was included to protect the analyses from the distorting effects of potential extreme outliers when relatively few colonies (9) per apiary were sampled, by only taking the median colonies generally representative of the apiary they came from. In ‘*Varroa*-free’ regions (Dunedin in 2012 and the Chatham Islands), 5 colonies were randomly selected from each apiary site for inclusion in the analyses.

Throughout this study, sample sizes refer to the number of colonies examined. Representative samples of bees and mites were collected from each colony as follows. For virus analysis, a sample of 40 adult bees was collected from a frame containing open brood and honey. In *Varroa*-infested colonies, phoretic mite samples were also collected from the sugar shakes. Honeybee and *Varroa* samples were flash-frozen in liquid nitrogen and held in a liquid nitrogen dry shipper until they could be transferred to a freezer and stored at −80°C. All subsequent steps were performed in the Department of Zoology at the University of Otago.

### RNA extraction

Any mites present on the adult bees in each sample were removed on dry ice. The head of each bee was then removed and stored separately for future analysis.

Bee samples were divided into two batches of 20 decapitated bees. The two batches were treated separately in all subsequent steps as extraction replicates, thus providing data in duplicates for each colony.

Each batch was placed in a plastic mesh extraction bag (Bioreba) and flash-frozen in liquid nitrogen before being reduced to powder using a pestle. This operation was repeated three times. GITC buffer (4 mL) was added and samples were homogenised. For each *Varroa* sample, pools of 10 mites were homogenized in 110 µL GITC buffer using the Bullet Blender 24 bead mill (Next Advance) and a 1∶10 weight-ratio of 1.4 mm stainless blend beads (Next Advance), shaking the samples for 2×1 min.

Total RNA was extracted from 100 µL of each homogenate following the RNEasy plant mini kit protocol (Qiagen), eluted in 50 µL nuclease-free water, aliquoted and stored at −80°C until further processing. Within each batch of 20–30 samples, one “blank” extraction was performed using only GITC buffer, to test for contamination. RNA yield, concentration and quality were measured using a NanoDrop ND-100 spectrophotometer (NanoDrop Technologies).

### cDNA synthesis

For each sample, 150 ng of total RNA was reverse-transcribed in 10-µL reaction volumes using random hexamer primers and the Superscript III VILO cDNA synthesis kit (Invitrogen), according to the manufacturer's protocol. Each reaction also contained 0.1 ng of synthetic RNA250 (Ambion), added to the reaction mix as a passive reference gene for evaluating the cDNA reaction efficiency for each RNA sample [82].

### qPCR assays

Real-time qPCR was performed using primers designed to detect seven honeybee viruses: acute bee paralysis virus (ABPV), black queen cell virus (BQCV), chronic bee paralysis virus (CBPV), deformed wing virus (DWV), Israeli acute paralysis virus (IAPV), Kashmir bee virus (KBV) and sacbrood virus (SBV). Two assays were performed for DWV, and an additional assay was run for the ABPV complex (ABPV, KBV and IAPV). Each sample was also assayed for β-actin as an internal reference gene [83] using intron-spanning primers and for the passive reference RNA250. The assay primers and performance parameters are given in supplementary Table S1 [64], [84].

The assays were run on an Mx3000P thermocycler (STRATAGENE) using Express SYBR GreenER qPCR SuperMix (Invitrogen) in 20-µL reaction volumes containing 3 µL of 1∶10 diluted cDNA template, and 0.2 µM of each primer.

The cycling parameters were an initial denaturation step at 95°C for 2 min, followed by 40 cycles of denaturation for 15 s at 95°C, annealing for 20 s at 58°C, and extension for 30 s at 72°C followed by fluorescence reading. The amplification was followed by a dissociation curve analysis of the PCR products by raising the temperature from 72°C to 95°C, in 0.5°C increments.

Positive controls and non-template controls were included on each plate. Plasmids of known concentration containing inserts for each target were used to generate external standards for absolute quantification, obtained from 10-fold serial dilutions. Each plate contained four different concentrations of each external standard covering 7 orders of magnitude.

### RT-qPCR data conversion, transformation and normalisation

The specificity of each PCR product was verified using melting curve analysis and electrophoresis. Samples were assigned positive for a target if their melting temperature was similar to the melting temperature of the positive controls and if they had a Cq value no greater than 35.

The Cq values were determined at the same fluorescence threshold for all plates and all targets. For each target RNA, the Cq values of the external dilution standards of all RT-qPCR runs were pooled and plotted against their corresponding log_10_[template]. The linear regression equations were used to estimate the absolute amounts of virus, RNA250 and β-actin RNA in each reaction. The regression slopes were used to calculate the amplification efficiencies (E) of the different assays using the following equation: *E_assay_ = 10^-1/slope^* [85] (Table S1).

For all positive samples, the absolute virus RNA abundances per bee were then calculated by multiplying the amount per reaction by the different reaction and extraction dilution factors, including the individual cDNA synthesis efficiency obtained through the RNA250 passive reference gene assay. Finally data were normalised to the corresponding sample β-actin titre and weighted by the average β-actin titre.

Virus titres were log transformed to account for the exponential distribution of the data. Because it is not possible to log transform zero values, samples considered as negative were assigned a hypothetical Cq value of 36, which was converted to theoretical virus titres as described above. These “negative virus titres” were averaged to obtain the titer detection threshold for each target.

Average viral titres were calculated only for positive samples. Two separate assays using two different primer pairs were run for DWV. Since no IAPV or ABPV were found throughout the study, the ABPV-complex assay could be used as a second assay for KBV.

To resolve discrepancies in the data, as determined from biological replicates or from different assays run for DWV and KBV, samples were run a second time. Discrepancies persisted amongst some biological replicates. 100% prevalence concordance was obtained between the two assays run for both DWV and KBV, showing that our assays gave stable results.

Virus prevalence was defined as the percentage of colonies displaying Cq values ≤35 for each viral target amongst each region included in the study (bees: n = 114; mites: n = 39). Virus titres were calculated as presented above, and analysed on positive samples only (DWV_bees_: n = 114; KBV_bees_: n = 56; CBPV_bees_: n = 62; BQCV_bees_ n = 208; SBV_bees_: n = 144; DWV_mites_: n = 37; KBV_mites_: n = 13; CBPV_mites_: n = 7; BQCV_mites_ n = 14; SBV_mites_: n = 12).

### Statistical analysis

All statistical analyses and figures were generated in the R environment (version 3.0.2). Bee colonies were considered as the individual in all tests.

Due to the nature of the experimental design and the multiple parameters recovered from each colony, the analyses had to be performed on observations that are not necessarily independent. The use of Linear Mixed-effect Models (LMM) and Generalized Mixed Models (GLMM) allowed us to account for the non-independence of colonies within one apiary and/or of extraction replicates used to quantify virus titres. LMM and GLMM were carried out using the R function *lmer* (package *lme4*), with the number of years of *Varroa* exposure as a fixed effect, and apiary identities, colonies within apiary identities and/or extraction replicates as random factors. The process of generating a P-value is not straightforward for LMM [86]. Therefore, we provided P-values estimated by Markov Chain Monte Carlo (MCMC) sampling, implemented in the R function *pvals.func* (package *languageR*) [87]. 95% confidence intervals (CI_95%_) were also provided as another tool for assessing significance of the fixed effect [88]. Prevalence data were analysed according to a binomial distribution, and levels of *Varroa* infestation according to a Poisson distribution (count data). GLMM performed on virus titres were run using a Gamma distribution. Virus titres were analysed after log_10_ transforming the data. GLMM gave a superior identification of significant and non-significant effects in the data than LMM, with greater resolution and reduced noise. This is due to the greater ability of GLMM to account for non-linearity in the data, such as the year-2 peaks observed for many of the virus titres. Post-hoc comparisons were performed using Bonferroni corrections, which is the most conservative correction to adjust for error arising from multiple comparisons.

Comparisons of pathogen prevalence across the sampling transect were carried out using Chi-Square tests. Interactions between viruses or between virus prevalence in mite samples and bee samples were assessed using Chi-Square tests. In the case tables of contingency built for expected prevalence contained values lower than 5, Yates corrections were applied to the Chi-Square tests.

The relationship between *Varroa* infestation levels and DWV virus titres in bee samples, as well as between DWV virus titres in bees and in *Varroa* samples, was inferred by running regression analysis on linear models. To compare the evolution of the pathogen titres, the average titre was calculated for each region and for each pathogen target. These five-point time series were compared using pairwise correlation tests with a Spearman correction.

Principal Component Analysis (PCA) allowed us to compare the relative weight of the different pathogen titres variables, and to assess the explanatory power of these variables on the clustering of the samples according to the *Varroa* infestation gradient. PCA was built using the function *dudi.pca* (Package *ade4*), after centering and scaling the data to account for scaling differences between variables.

## Supporting Information

Figure S1 **Comparisons and correlations of pathogen titres in honeybee and** ***Varroa*** **samples.** (**A**) Pathogen titres in bee samples according to the number of years of confirmed exposure to *Varroa*. Error bars indicate the SEM. Comparisons were made between the dynamic changes of mean viral titres and mean infestation rates recorded for *Varroa*. Significant correlations between KBV and BQCV titre distributions and *Varroa* infestation rate distribution were identified (Correlation tests, p<0.05). (**B**) Correlation of overall DWV viral titres in bee samples versus overall *Varroa* infestation levels. The regression is significant (LM, F_1,54_ = 17.63, p<0.01, n = 56), but the degree of correlation is not high (r^2^ = 0.25). (**C**) Correlation of DWV viral titres in bee samples versus DWV viral titres in *Varroa* samples. The regression is highly significant (LM, F_1,28_ = 27.81, p<0.01, n = 31), and shows a large degree of correlation (r^2^ = 0.5).
(TIF)

Table S1 **Primer sequences and performance indicators of the RT-qPCR assays run for the different honeybee viruses and the *Apis mellifera* and *Varroa destructor* internal reference genes.**
(DOCX)
