# Supplementary figures and images for: On the Front Line: Quantitative Virus Dynamics in Honeybee (Apis mellifera L.) Colonies along a New Expansion Front of the Parasite Varroa destructor
Source: PLoS Pathog. 2014 Aug 21;10(8):e1004323. doi: 10.1371/journal.ppat.1004323 (PMC4140857; doi:10.1371/journal.ppat.1004323)

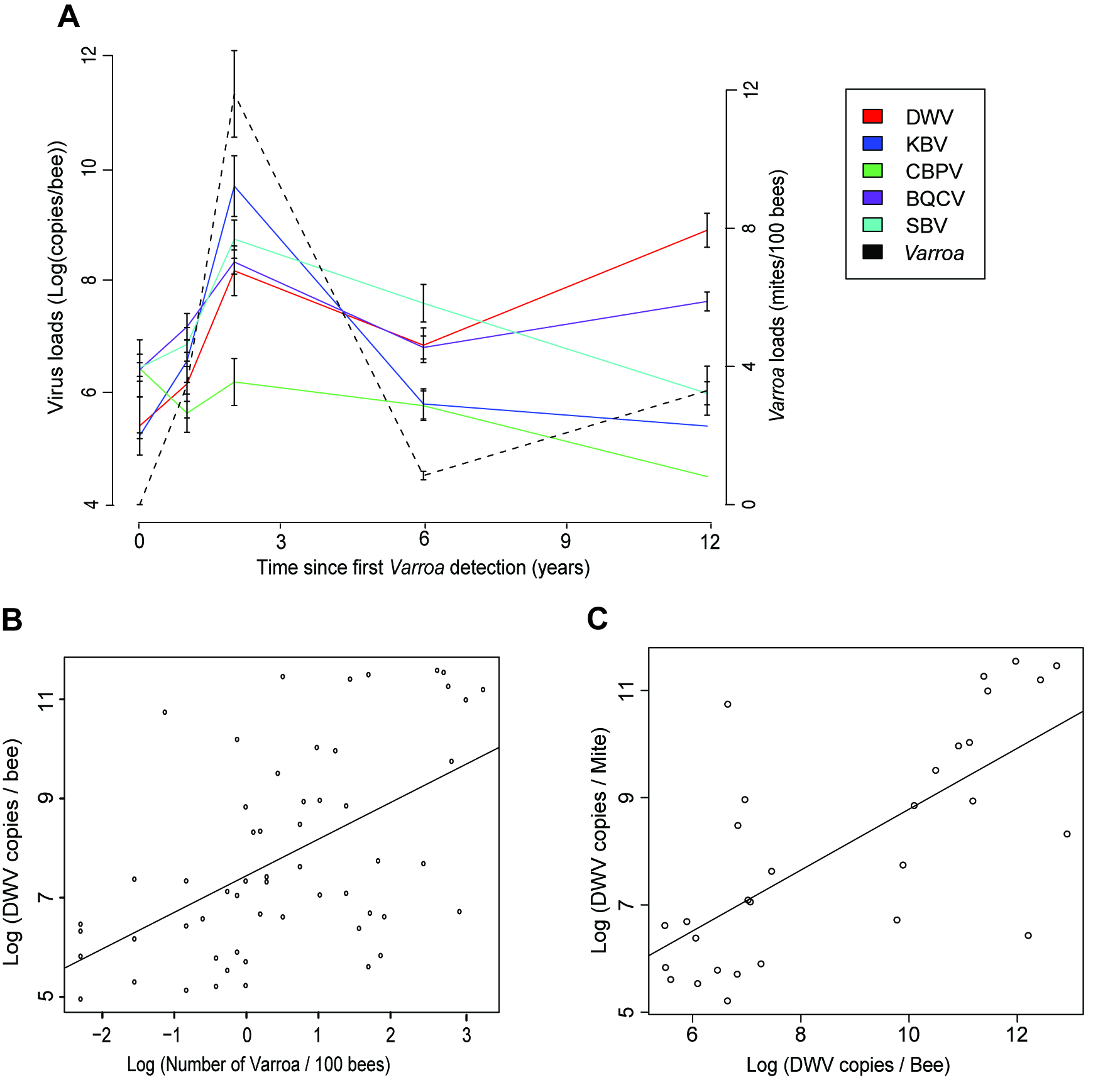

Supplement: Figure S1 — Comparisons and correlations of pathogen titres in honeybee and Varroa samples. (A) Pathogen titres in bee samples according to the number of years of confirmed exposure to Varroa. Error bars indicate the SEM. Comparisons were made between the dynamic changes of mean viral titres and mean infestation rates recorded for Varroa. Significant correlations between KBV and BQCV titre distributions and Varroa infestation rate distribution were identified (Correlation tests, p<0.05). (B) Correlation of overall DWV viral titres in bee samples versus overall Varroa infestation levels. The regression is significant (LM, F1,54 = 17.63, p<0.01, n = 56), but the degree of correlation is not high (r2 = 0.25). (C) Correlation of DWV viral titres in bee samples versus DWV viral titres in Varroa samples. The regression is highly significant (LM, F1,28 = 27.81, p<0.01, n = 31), and shows a large degree of correlation (r2 = 0.5). (TIF) [file ppat.1004323.s001.tif]
