# Supplementary material for: On the Front Line: Quantitative Virus Dynamics in Honeybee (Apis mellifera L.) Colonies along a New Expansion Front of the Parasite Varroa destructor
Source: PLoS Pathog. 2014 Aug 21;10(8):e1004323. doi: 10.1371/journal.ppat.1004323 (PMC4140857; doi:10.1371/journal.ppat.1004323)
Supplement: Table S1 — Primer sequences and performance indicators of the RT-qPCR assays run for the different honeybee viruses and the Apis mellifera and Varroa destructor internal reference genes. (DOCX) [file ppat.1004323.s002.docx]

| **Target** | **Primers** | **Sequence (5’ – 3’)** | **Size (bp)** | **E** | **r^2^** | **Tm (°C)** |
| --- | --- | --- | --- | --- | --- | --- |
| DWV | DWV-F8688 | GGTAAGCGATGGTTGTTTG | 143 | 2.014 | 0.982 | 79.5 |
|  | DWV-B8794 | CCGTGAATATAGTGTGAGG |  |  |  |  |
| DWV | DWV-F1425 | CGTCGGCCTATCAAAG | 417 | 2.001 | 0.997 | 82 |
|  | DWV-B1806 | CTTTTCTAATTCAACTTCACC |  |  |  |  |
| ABPV complex | AKIV-F6677 | GATACCCCCATGGCTC | 66 |  |  | 79 |
|  | KIABPV-B6707 | CTGAATAATACTGTGCGTATC |  |  |  |  |
| ABPV | ABPV-F6548 | TCATACCTGCCGATCAAG | 197 |  |  | 82.1 |
|  | KIABPV-B6707 | CTGAATAATACTGTGCGTATC |  |  |  |  |
| KBV | KBV-F6639 | CCATACCTGCTGATAACC | 200 | 1.870 | 0.995 | 82.5 |
|  | KIABPV-B6707 | CTGAATAATACTGTGCGTATC |  |  |  |  |
| IAPV | IAPV-F6627 | CCATGCCTGGCGATTCAC | 203 |  |  | 82.6 |
|  | KIABPV-B6707 | CTGAATAATACTGTGCGTATC |  |  |  |  |
| BQCV | BQCV-qF7893 | AGTGGCGGAGATGTATGC | 294 | 2.015 | 0.993 | 81.6 |
|  | BQCV-qB8150 | GGAGGTGAAGTGGCTATATC |  |  |  |  |
| CBPV | CBPV1-qF1818 | CAACCTGCCTCAACACAG | 296 | 1.921 | 0.992 | 86.1 |
|  | CBPV1-qB2077 | AATCTGGCAAGGTTGACTGG |  |  |  |  |
| SBV | SBV-qF3164 | TTGGAACTACGCATTCTCTG | 335 | 1.884 | 0.995 | 81.6 |
|  | SBV-qB3461 | CTCTAACCTCGCATCAAC |  |  |  |  |
| β-actin mRNA | Am-actin2-qF | CGTGCCGATAGTATTCTTG | 271 | 1.886 | 0.988 | 87.2 |
| (*A.mellifera*) | Am-actin2-qB | CTTCGTCACCAACATAGG |  |  |  |  |
| β-actin mRNA | Vd-actin-qF | CGACGGTCAGGTCATCAC | 243 | 2.009 | 0.991 | 84.2 |
| (*V.destructor*) | Vd-actin-qB | GTTGAGGGAGCCAAAGAGG |  |  |  |  |
| RNA250 (Ambion) | RNA250-F | TGGTGCCTGGGCGGTAAAG | 227 | 2.018 | 0.995 | 86.7 |
|  | RNA250-R | TGCGGGGACTCACTGGCTG |  |  |  |  |

Table S1. Primer sequences and performance indicators of the RT-qPCR assays run for the different honeybee viruses and the Apis mellifera and Varroa destructor internal reference genes
